# Supplementary material for: “Each moon we come to weigh the pregnancy:” Exploring the experience of group antenatal care processes in Benin and their contributions to self-efficacy
Source: PLOS Glob Public Health. 2026 Jun 5;6(6):e0004851. doi: 10.1371/journal.pgph.0004851 (PMC13240911; doi:10.1371/journal.pgph.0004851)
Supplement: S1 Appendix — (DOCX) [file pgph.0004851.s001.docx]

**Appendix 1. Group antenatal care processes and their intended effect on**

**self-efficacy**

| **Group ANC (G-ANC) session framework adapted from Grenier, et al 2022** | **G-ANC process used in Benin** | **How process promotes self-efficacy (theorized)** |
| --- | --- | --- |
| **ASSESS AND CHECK-IN** | | |
| **Self-assessments** at the start of every session | - Women take their blood pressure (BP) with automated devices and weigh themselves, often in pairs or their “sister-up” groups. - Participants write the readings on a laminated self-assessment card to show the provider. Women who are unable write are assisted by other women or the provider. - Self-assessment cards include simple illustrations of danger signs during pregnancy. Women circle any picture if they are experiencing any of these symptoms and crossing out those they do not have. - Providers move around to help women (e.g., adjust BP cuff, change BP cuff size) and answer questions. | - Engaging women in their own health care creates comfort and familiarity with clinical procedures and increases ownership over their health. - Vicarious experience of watching other women take weight and BP builds confidence to perform new skills. - Self-assessment of danger signs validates their experience and helps apply the knowledge of specific danger signs. - Illiterate women often express that “writing” is new for them and they gain confidence by holding a marker and circling or crossing out each picture. |
| **Consultation:** Brief one-on-one examination with a provider | - Provider reviews self-assessment cards, conducts clinical exam, and answers any questions. | - Answering questions participants ask individually creates an opportunity for verbal persuasion. |
| **REVIEW, SHARE, LEARN AND PRACTICE** | | |
| **Circle up (opening ritual):** Come together and be present | - Brief activity to connect the group and relax/de-stress participants before beginning the session. | - Not designed to increase self-efficacy, but for bonding with peers which creates the environment for support. Relaxation can also create the conditions for improved self-efficacy if women feel less stressed or overwhelmed. |
| **Review** previous session and successes/challenges | - Provider asks participants to briefly review the previous session’s topics. Any participants who missed last session get updated. | - Reminds and reinforces knowledge and practice. - Empowers participants to share with their peers which validates their knowledge and builds confidence. |
| **Introduce today’s topics** with a story or activity | - Brief stories, games and role plays to engage women on a key topic for the day’s session. - For example, the “Who, Like Me...?” game has a woman stand in the middle of the circle and share something, e.g., *Who like me slept under a bed net last night?* Women in the circle who agree stand up, and one volunteers to go to the middle of the circle and make another statement. | - Validates women’s lived experiences and knowledge. - Connects women with peers who have similar experiences, and demonstrates for others that their peers are practicing healthy behaviors. |
| **Facilitate discussion** about gestationally appropriate topics using illustration cards. Share knowledge, agree on actions, discuss barriers and solutions. | - Each session covers 1-2 key topics. - Topics are introduced using 5-8 pictorial cards, with simple illustrations designed for non-literate audiences. Topics relate to “preventing problems” or “recognizing problems and taking action.” - Discussions are facilitated by providers through a series of open-ended questions. The process for each card first builds on women’s knowledge and experiences, then asks about challenges to practicing key behaviors. Each card ends with building group consensus to take action, i.e., *Can we agree that these are all serious problems and we will seek help at a health facility if we experience any danger signs?* *Can we agree we will teach our families about these danger signs and ask for help to get to a facility if needed?* | - Promotes behavior change grounded in the Home-Based Life Saving Skills (HBLSS) methodology (Dynes, et al, 2011). - Discussion between provider and participants and among participants creates opportunities for verbal persuasion. |
| **Practice using and sharing new knowledge** | - Brief activity as a large group to apply knowledge, such as a game to practice identifying danger signs, versus other common complaints/discomforts of pregnancy which are bothersome (e.g., reflux/heartburn) but not dangerous. - Participants take a dose of Intermittent Preventive Treatment of malaria in pregnancy (IPTp) under directly observed treatment. | - Engages women in a fun, low-stakes interaction so they can apply knowledge. Discussion can be lively and use verbal persuasion to help correct misinformation. - Vicarious experience of watching other women take IPTp normalizes this behavior and builds confidence. |
| **REFLECT, PLAN AND SOCIALIZE** | | |
| **Sister-up** and plan to add a new behavior | - Each woman shares how well they did on the 2-3 small, doable actions they identified in the previous session and intended to practice. - Women pair up to make individual action plans based on the day’s discussion. - Each woman shares 2-3 small doable actions they want to take on their own before the next meeting, and talk through how they will address any challenges. | - Smaller groups within the larger one provide an opportunity for each woman to engage and provide encouragement to others. - Acting on their own to take small, doable actions helps women apply new knowledge after the meeting and experience performance accomplishments. - Review creates accountability to the group as a motivator to take action. |
| **Review key discussion points** and individual action plans | - Provider asks the sister-up groups to share their discussions, and reviews key takeaways from the meeting. | - Sharing can create a sense of social support and accountability to the group. |
| **Prepare for next session** | - Provider shares the date, time, and topics of the next meeting. Provider also asks the women, *Can you help remind each other?* - For women who are reachable by phone, providers call or text participants a few days before the next session to remind them to come. | - Participants reminding one another creates social support and allows an opportunity for verbal persuasion to continue attending G-ANC. - Builds curiosity/interest and peer support to return for the next meeting. |
| **Closing ritual** | - Brief activity to connect the group before ending the session, such as an affirmation or song. | - Builds a sense of community within the group. - Positive emotional state created gives women greater pleasure-based motivation to return to G-ANC. |
| **Socialize** | - At the end of the session, women are encouraged to stay in the group and socialize. Often if there are refreshments available, they are distributed and eaten/shared at this time. | - Builds social capital through peer support and creates a sense of community within the group. - Not specific to self-efficacy for key behaviors but may encourage women to return for more G-ANC meetings, i.e., more ANC contacts. |
