# Supplementary material for: “Each moon we come to weigh the pregnancy:” Exploring the experience of group antenatal care processes in Benin and their contributions to self-efficacy
Source: PLOS Glob Public Health. 2026 Jun 5;6(6):e0004851. doi: 10.1371/journal.pgph.0004851 (PMC13240911; doi:10.1371/journal.pgph.0004851)
Supplement: S2 Appendix — (DOCX) [file pgph.0004851.s002.docx]

**Appendix 2. Comparison of individual antenatal Care (ANC) and group antenatal care (G-ANC) as implemented in Benin**

| **Antenatal Care Components*** | **Individual ANC: Control Arm** | **Group ANC: Intervention Arm** |
| --- | --- | --- |
| 1. **Clinical care** by a health care   provider in a private space | Yes | Yes |
| - Initial history taking and physical exam | Yes | Yes, completed at first individual ANC visit. At this first visit, eligible pregnant women (based on their gestational age) could decide to enroll in G-ANC to receive subsequent ANC in a group setting. |
| - Patient assessment, clinical exam, review of medical records and lab results | Yes | Yes, examinations were generally done behind privacy screens in the group meeting space. Where meetings occurred in tents due to space constraints, individuals examinations took place in health facility exam rooms. |
| - Provision of vaccinations, medicines (e.g., IPTp), rapid tests | Yes. IPTp and other interventions provided outside of ANC appointment. | Yes, IPTp provided via DOTS during G-ANC meetings; other interventions provided outside of G-ANC meeting. |
| - Patient-led self-assessments: blood pressure, weight, danger signs and symptoms self-reporting | No | Yes |
| - Referral for other services (e.g., specialist, ultrasound, lab, pharmacy) | Yes | Yes |
| 1. **Counseling and health information** on prevention; healthy behaviors; danger signs; common problems during pregnancy, childbirth and postnatal period; promotion of health facility use (especially for childbirth) | Yes, by provider during individual visit as time permits. | Yes, through facilitated, highly participatory discussions lasting approximately 40-60 minutes. |
| - Counseling from a consistent health care provider | No, any qualified ANC provider may see and counsel women | Yes, one or two trained providers consistently facilitated group meetings and developed relationships with women. |
| - Pictorial health booklet with key messages to take home | No | Yes |
| 1. **Peer support** | No | Yes, women with similar due dates interacted and developed relationships with other pregnant women at G-ANC meetings. |

Components of antenatal care (ANC) adapted from: World Health Organization. (2016). *Pregnancy, childbirth, postpartum and newborn care: a guide for essential practice – 3rd ed*.
