# Supplementary material for: “Each moon we come to weigh the pregnancy:” Exploring the experience of group antenatal care processes in Benin and their contributions to self-efficacy
Source: PLOS Glob Public Health. 2026 Jun 5;6(6):e0004851. doi: 10.1371/journal.pgph.0004851 (PMC13240911; doi:10.1371/journal.pgph.0004851)
Supplement: S3 Appendix — (DOCX) [file pgph.0004851.s003.docx]

**Appendix 3. Health Facility Characteristics Considered for Purposive Sampling**

| **Health Zone** | **Health Center Implementing**  **G-ANC** | **Classification**   (Urban, Peri-urban, Rural) | **Volume of ANC Clients**   (Low, High) | **G-ANC Retention Rate**   (% women attending all 5 meetings) | **Number of**  **G-ANC Enrollees**   (Mar 2021-Mar 2022) |
| --- | --- | --- | --- | --- | --- |
| AS | CS KPANROUN | Rural | Low | 16% | 95 |
|  | CS GANVIÉ | Rural | Low | No data | 45 |
|  | CS OUÈDO | Peri-urban | High | 81% | 285 |
|  | CS ZINVIÉ | Peri-urban | High | 55% | 98 |
|  | CS DEKOUNGBÉ | Peri-urban | High | 31% | 83 |
|  |  |  |  |  |  |
| ATZ | CS TANGBO DJEVIE | Rural | High | 23% | 80 |
|  | CS SEDJE-DENOU | Rural | Low | 36% | 34 |
|  | CS AGON | Rural | Low | 46% | 127 |
|  | CS HOUEGBO | Rural | Low | 49% | 100 |
|  | CS SOYO | Rural | Low | No data | 56 |
|  | CS SEHOUE | Rural | Low | 27% | 143 |
|  | CS DODJI-BATA | Rural | High | 9% | 106 |
|  | CS TOGOUDO | Rural | Low | 75% | 70 |
|  |  |  |  |  |  |
| OKT | CS TORI GARE | Rural | Low | 68% | 124 |
|  | CS SAVI | Peri-urban | High | 51% | 107 |
|  | CS AVAME | Rural | Low | 23% | 90 |
|  | CS TORI-CADA | Rural | Low | 65% | 141 |
|  | CS TORI-BOSSITO | Peri-urban | Low | 43% | 89 |
|  | CS KPOMASSE | Peri-urban | Low | 30% | 94 |
|  | CS KPOVIE | Rural | Low | 63% | 91 |
|  |  |  |  |  |  |
|  |  | Selected for qualitative study |  |  |  |
