# Supplementary material for: “Each moon we come to weigh the pregnancy:” Exploring the experience of group antenatal care processes in Benin and their contributions to self-efficacy
Source: PLOS Glob Public Health. 2026 Jun 5;6(6):e0004851. doi: 10.1371/journal.pgph.0004851 (PMC13240911; doi:10.1371/journal.pgph.0004851)
Supplement: S4 Appendix — (DOCX) [file pgph.0004851.s004.docx]

Appendix 4

# Tool 05: Focus group with pregnant women and women who have recently given birth

## Objectives of the tool

1. To identify factors that affect adherence to intermittent preventive treatment for malaria during pregnancy, and how participation in group antenatal care (GANC) may affect this.
2. To describe the experience of participating in Group ANC sessions for pregnant women.
3. To assess the acceptability of additional questions on malaria-related behaviors at the first antenatal visit as part of malaria surveillance in pregnant women.

The questions in this tool are organized into modules, each with a series of numbered questions. The interviewer should aim to ask a few questions from each module. However: 1) It is not necessary to always ask the questions in the order they are asked. The order in which the questions are asked may change, in order to follow up on the respondent's previous responses. 2) Some questions may be omitted, if the content is covered in the responses to other questions, or if the question is not relevant to the women in the focus group.

## Selection of participants

The study is implemented in three health zones of the Atlantic department. We propose to select health centers with high completion rates for Group ANC, and also health centers with an average completion rate. All women participating in these focus groups must have participated in at least three group antenatal consultation sessions. The proposed sample is presented in the table below.

| Health zone | AS = Abomey-Calavi-So-Ava | | ATZ = Allada-Toffo-Zè | | OKT = Ouidah-Kpomassè-Tori Bossito | |
| --- | --- | --- | --- | --- | --- | --- |
| Health center | CS Kpanroun | CS Ouèdo | CS Tangbo Djevie | CS Togoudo | CS Tori Station | CS Savi |
| 8 to 12 women per FG  N=6 FG | 1 focus group | 1 focus group | 1 focus group | 1 focus group | 1 focus group | 1 focus group |

We aim for a minimum of 8 women participating in each focus group. If fewer than 8 women attend, then the interviewers should ask the questions contained in the tool. However, this will not count towards the sample size of six focus groups. There may be ambiguous situations. If the group starts with 6 participants and two more women arrive after 15 minutes, then this could count towards the sample size of six focus groups. Women will be provided with transport costs, as they will need to travel out of their villages.

## Module 1 – Focus Group Information

| Evaluation of the impact of group antenatal consultation (Group ANC) on Intermittent Preventive Treatment (IPT) of Malaria in pregnant women and the use of the population of pregnant women as sentinels for malaria surveillance in Benin.  Investigators - Prof. Aurore Ogouyèmi-Hounto, Dr. Blaise Guézo-Mevo, Dr Julie Gutman  Protocol Identifiers: 1) CDC: 7254; 2) BENIN: 43/MS/DRFMT/CNERS/SA | |
| --- | --- |
| Focal group code |  |
| Instrument number | Tool 05 |
| Instrument version/date | Saturday May 14, 2022 |
| Method | Focus group |
| Language | French / Fon |
| Study Participants | Pregnant women and women who have recently given birth |
| Consent form for the method | Consent Form C |
| 1. Date of the focus group |  |
| 1. Health zone | [ ] 1. Abomey-Calavi-So-Ava  [ ] 2. Allada-Toffo-Zè  [ ] 3. Ouidah-Kpomassè-Tori-Bossito |
| 1. Health facility where women attended antenatal consultations |  |
| 1. Number of women participating in the focus group |  |
| 1. Name of focus group facilitator |  |
| 1. Names of note takers for the focus group |  |
| 1. Focus group start time |  |
| 1. Focus group end time |  |

## Module 2 - Extracting information from women's files

This information will need to be entered for all women participating in the focus group into a table. The rows will be variables 201 to 209. The columns will be the women participating in the focus group discussion, one column for each woman. The data from this sheet will be entered into an Excel spreadsheet.

| 1. Age of the woman |  |
| --- | --- |
| 1. Marital status | 01 - NEVER MARRIED  02 - MARRIED/LIVING TOGETHER  03 - WIDOW  04 - DIVORCED/SEPARATED |
| 1. How many births have you had in total? | NUMBER OF BIRTHS \|____\|____\| |
| 1. How many times in total did you attend prenatal visits during your last pregnancy? | NUMBER OF TIMES ____  DON'T KNOW / DON'T REMEMBER 98 |
| 1. Have you been offered group prenatal care? That is, care where all your visits would be with the same group of women? | [ ] 1. YES  [ ] 2. NO |
| 1. If offered, have you participated in group prenatal counseling sessions? | [ ] 1. YES  [ ] 2. NO |
| 1. How many group prenatal counseling sessions has the woman attended? |  |
| 1. How many times did the woman receive intermittent preventive treatment with Fansidar? |  |
| 1. Confirm that the woman has consented   (If NO, do not continue with the FGD) | [ ] 1. YES  [ ] 2. NO |

## Information for the investigator

Remind participants that participation is voluntary, they can choose not to answer any questions. Explain to participants that you are interested in learning more about the well-being of pregnant women in this area. Explain why they were chosen for the focus group and that their perspective is especially important to you. Emphasize that you want to learn from them, that there are no right or wrong answers and that they can stop at any time. Let participants talk about what they think is important. Remind them that everything you talk about will be kept confidential.

## Module 3 – Most recent pregnancy

We are extremely interested in your pregnancy experiences and your perspective is particularly important to us. We would love to learn from you and there are no right or wrong answers. Please stop at any time if you have something to say. We will not record your name and anything you talk about will be kept confidential.

1. In this community, when would a woman tell her husband that she is pregnant? When would a woman tell other family members? When would she tell neighbors, friends, and other community members?
2. Now I would like to ask you about health during pregnancy. What do you think are the important things to do to stay healthy during pregnancy?
   1. What do you do to protect your health when you are pregnant?
   2. What do you do to ensure your baby is born healthy?
3. What particular care does a woman need when she is pregnant? (ANC, diet, chores, medications, etc.)
   1. Where do you get this care?
   2. Besides yourself, who helps decide about your care during pregnancy?
4. What happens if this person or people are absent or unreachable?

## Module 4 – Group Prenatal Consultation Sessions

1. During this last pregnancy, did you attend any meetings during prenatal consultations where pregnant women met in groups?
   1. Where did your group meet?
   2. Can you describe to me what happened during these group meetings?
2. What did you like about participating in group prenatal consultations? What did you not like?
3. What makes it easier for you to participate in these group meetings? Why?
4. What makes it difficult for you to participate in these group meetings? Why?
5. How satisfied were you with the care you received when you attended these group meetings with other pregnant women?
6. What did you learn from attending these group meetings?
7. Did you feel more confident about taking care of yourself during pregnancy and preparing for childbirth after attending these group meetings?
8. What did you learn about the different pills and medications to take during pregnancy during these group meetings?
9. Do you think you were more likely to take the medications offered to you by health providers after attending these group meetings? Why?
10. Some of you here today may have had a previous pregnancy where you did not attend group meetings. What differences do you see between attending group meetings during pregnancy and coming just for individual visits?
11. What are your suggestions for improving group meetings?
12. If your sister or a friend were pregnant and asked you if she should attend these group meetings, what would you advise her? Why would you advise her to do this?
13. Did you attend the individual antenatal consultations, after having attended [one or two] group antenatal consultation sessions? Did you take the malaria tablets during these last individual visits?
14. What difficulties did you have in taking the malaria treatment?

## Module 5 – Malaria during pregnancy

1. Now I would like you to tell me about pregnant women and malaria. How important is malaria as a problem for pregnant women here?
2. Some people say that getting malaria while pregnant is the same as getting malaria at any other time. Some people say that getting malaria is more dangerous when you are pregnant. What do you think?
3. What have you done/what are you doing to avoid getting malaria?
4. Some health facility providers give pregnant women tablets (Fansidar/SP) to prevent malaria. Did the providers at your health facility give you tablets to prevent malaria?
5. What was your experience with these tablets? What made you decide to take the tablets/not take the tablets?
6. Women should take these tablets 3 or 4 times during pregnancy to prevent malaria. But many women take the tablets only once or not at all. What are the reasons why many women do not take the tablets at all, or take them only once, instead of 3 or 4 times?
7. When providers at a health facility tell you to take medicine, do they give you the medicine they want you to take? Is it free or do you have to pay? If you have to pay, how much does it cost? Is it an affordable price? (If not, what would be an affordable price?)
8. When health facility staff tell you to take SP/Fansidar, do they have it available? (always/sometimes/never) If they do not have it, where do you get it?

## Module 6 - Family planning

1. Now I would like to ask you about family planning, any advice you may have received during your prenatal visits, and future plans for using family planning. Have you used any family planning methods in the past, prior to this pregnancy? What methods have you used?
2. During group meetings, did the provider discuss family planning? What family planning methods did the provider discuss with the group?
3. As a result of these group meetings, did any of you decide to do anything to avoid getting pregnant after giving birth?

## Module 7 – Malaria surveillance during antenatal consultations

1. How would you like to have spent 10 minutes during your first antenatal visit answering questions about malaria prevention at home. Questions might include taking medicines to prevent malaria, sleeping under mosquito nets, and treating children with fever?
2. Do you think this improved the care you received? If so, how?
3. How do you feel about the time you spend at antenatal care? Is the time spent too long or too short? Would it make a difference to you to spend 10 more minutes answering these questions about malaria at your first antenatal care?
