# Supplementary material for: “Each moon we come to weigh the pregnancy:” Exploring the experience of group antenatal care processes in Benin and their contributions to self-efficacy
Source: PLOS Glob Public Health. 2026 Jun 5;6(6):e0004851. doi: 10.1371/journal.pgph.0004851 (PMC13240911; doi:10.1371/journal.pgph.0004851)
Supplement: Attachment — Submitted filename: PLOS Manuscript Review PGPH-D-25-01395.docx [file pgph.0004851.s006.docx]

Manuscript: PGPH-D-25-01395

Introduction

- To improve the flow, I recommend discussing outcomes (lines 59-64) and then introducing the theory of change (lines 57-59) as a mechanism for why we see these outcomes. For readers who are unfamiliar with the theory of change proposed by Grenier et al., I think it would be beneficial to elaborate on how G-ANC increases self-efficacy in 1-2 sentences. This then ties into you introducing/defining self-efficacy in lines 65-74.
- I would like to see more discussion in the paragraph about G-ANC processes (75-79). You correctly specify that “theoretically” the processes are taking place, and you lay them out well in your Table 1 and Figure 1. I feel like there is a real opportunity here to highlight the gap in the literature that you are filling with this manuscript. Not many other G-ANC studies, particularly in LMICs measure self-efficacy, but given its crucial role in the theory of change, this paper is a great value add to the literature.
- In lines 80-85, I recommend parsing out the aims of the parent study and the aims of this paper, specifically. In their current form, the wording of these three do not directly match the results.

Methods

- For clarity, I would discuss the quant and qual aspects of the paper in the same order. For example, qual aims then quant aims, qual methods then quant methods, qual analysis, then quant analysis, etc. – there seems to be some switching back and forth.
- You mention that the focus groups were conducted in Fongbe and transcribed to French (lines 120-121) – were they then translated to English for analysis? It seems like the deductive analysis was using English word codes – was the coding done in English or French?
- In your study limitations section, you state that interviews and focus groups were not designed to investigate the effect of G-ANC on women’s self-efficacy, so I would consider re-framing this as a secondary qualitative analysis.
- In lines 133-134, you briefly discuss the software used for quantitative data collection and analysis, but you do not discuss what analyses were performed. For example, what statistical tests were used to get the p-values in Table 3?

Results

- Lines 148-163 feel a bit out of place to me. You reference Table 2 at the end of line 150, but Table 2 has no mention of social support, stressful situations, or self-advocacy, which is what the preceding sentences are referring to. Are these supposed to be overall insights into women’s experiences (related to Aim 1)? If so, incorporation of themes would be helpful. I am not quite getting the link between what is discussed here and self-efficacy, if it is supposed to be connected.
- The content domains of self-efficacy and the sources of efficacy expectations were very clear.
- The connection between the quant survey findings and the qualitative findings is a bit unclear for some measures. It may be that there needs to be more information in the introduction section about how certain survey indicators are related to self-efficacy. How do the outcomes in line 240-244 relate to self-efficacy? Additionally, you present data on the statement: “I was given the opportunity to ask questions about family planning methods.” To me, this more so speaks to provider quality and fidelity to the curriculum and less about self-efficacy. I was also curious why there was a quantitative focus on family planning when this did not come up at all in any of the qualitative data?
- I do not see Table 4 referenced in the text. The data presented in Table 4 is very interesting, but I am not quite getting the connection to self-efficacy. Perhaps it is related to one of the other aims? Overall, I would like to see the aims, the methods for each aim, and the results for each aim more clearly outlined.

Discussion

- The discussion could also be reorganized to reflect the findings of each aim. I recommend starting out with exactly what you found and then related it back to the theory of change and other literature. You were going back and forth between the qual and quant findings, which was a bit confusing.
- Great job connecting to other studies in LMICs! However, I think you could be a bit more detailed. For example, in lines 310-312, you state “Studies in Nepal, Senegal, Bangladesh, and Rwanda had similar findings.” Similar findings in that women had more positive experiences or that they would prefer G-ANC over individual ANC. Each study words their findings / measure these concepts in different ways, so you may want to explicitly discuss what each found.

Conclusion

- The first sentence (line 352) makes me think assess acceptability was supposed to be a main aim of this paper. Maybe that is a part of you aim 1? If this is the case, maybe aim 1 should be reworded and the title changed to reflect the focus beyond self-efficacy.
