# Supplementary material for: “Each moon we come to weigh the pregnancy:” Exploring the experience of group antenatal care processes in Benin and their contributions to self-efficacy
Source: PLOS Glob Public Health. 2026 Jun 5;6(6):e0004851. doi: 10.1371/journal.pgph.0004851 (PMC13240911; doi:10.1371/journal.pgph.0004851)
Supplement: Attachment — Submitted filename: PGPH-D-25-01395R1_Response_reviewers_2025-09-26.docx [file pgph.0004851.s007.docx]

Response to reviewers

Date: Friday, September 26, 2025

PGPH-D-25-01395R1

“Each moon we come to weigh the pregnancy:” Exploring the experience of group antenatal care processes in Benin and their contributions to self-efficacy

Corresponding author: Prof. Peter Winch, Johns Hopkins University

Responses are highlighted in yellow below. Thank you. Peter Winch

From: em.pgph.0.95f5b8.b339d534@editorialmanager.com <em.pgph.0.95f5b8.b339d534@editorialmanager.com> On Behalf Of PLOS Global Public Health

Sent: Monday, September 8, 2025 5:39 AM

To: Peter Winch <pwinch@jhu.edu>

Subject: PLOS Global Public Health: Your submission PGPH-D-25-01395R1 - [EMID:726bc9783ef8eebf]

PGPH-D-25-01395R1

“Each moon we come to weigh the pregnancy:” Exploring the experience of group antenatal care processes in Benin and their contributions to self-efficacy Professor Peter John Winch

Dear Dr. Winch,

Thank you for submitting your manuscript entitled "“Each moon we come to weigh the pregnancy:” Exploring the experience of group antenatal care processes in Benin and their contributions to self-efficacy" to PLOS Global Public Health. Your manuscript files have been checked in-house, but before we can proceed we need you to address the following issues:

1. We note that your Data Availability statement states the following: "The behavioral data used to generate the quantitative tables for this article are available here: https://qdr.syr.edu/. The relevant quantitative data are presented in the tables." Please advise us if this statement and the provided repository is acceptable."

However, we note that the link provided leads to a general home page. At this time, please provide direct links to the data used for this study, or any relevant dataset titles and/or accession numbers needed to access the data at this repository.

- Upon further investigation, we found that we are not eligible to upload our data to the identified repository. All quantitative data are included in the tables.

2. Thank you for providing the requested questionnaire for global inclusivity. However, we note that the questionnaire has been submitted as an ""other"" file type. Kindly resubmit the global inclusivity questionnaire as Supporting file.

🡺 Done

3. We have amended your Competing Interest statement to comply with journal style. We kindly ask that you double check the statement and let us know if anything is incorrect.

🡺 All is correct

4. Your current Financial Disclosure states, “This study was made possible by the generous support of the American people through the United States Agency for International Development (USAID) under the terms of contract 7200AA18C00014.”. However, your funding information on the submission form indicates that you received funding from “President's Malaria Initiative”. Please indicate by return email the full and correct funding information for your study and confirm the order in which funding contributions should appear. Please be sure to indicate whether the funders played any role in the study design, data collection and analysis, decision to publish, or preparation of the manuscript.

🡺 This study was made possible by the generous support of the American people through the President's Malaria Initiative under the terms of United States Agency for International Development (USAID) contract 7200AA18C00014.

5. Please upload all main figures as separate Figure files in .tif or .eps format only. For more information about how to convert and format your figure files please see our guidelines:

🡺 Done

6. Please ensure that the Title in your manuscript file and the Title provided in your online submission form are the same.

🡺 Done
