# Supplementary material for: “Each moon we come to weigh the pregnancy:” Exploring the experience of group antenatal care processes in Benin and their contributions to self-efficacy
Source: PLOS Glob Public Health. 2026 Jun 5;6(6):e0004851. doi: 10.1371/journal.pgph.0004851 (PMC13240911; doi:10.1371/journal.pgph.0004851)
Supplement: Attachment — Submitted filename: Response to Reviewers - Letter - 26 March 2026.docx [file pgph.0004851.s008.docx]

The Editors

PLOS Global Public Health

March 26, 2026

Dear Editorial Board:

Once again, we would like to thank the reviewers for their thoughtful feedback on this research. We feel their input has helped us to strengthen the manuscript substantially. Our responses to each of the points raised follow below, and we hope that this version will now meet the journal's rigorous publication criteria.

We have uploaded a Revised Manuscript with Track Changes along with an unmarked Manuscript file.

Sincerely,

Peter J. Winch

Corresponding Author

[Pwinch@jhu.edu](mailto:Pwinch@jhu.edu)

<<<>>>

Comments to the Author
1. If the authors have adequately addressed your comments raised in a previous round of review and you feel that this manuscript is now acceptable for publication, you may indicate that here to bypass the “Comments to the Author” section, enter your conflict of interest statement in the “Confidential to Editor” section, and submit your "Accept" recommendation.

Reviewer #1: (No Response)

Reviewer #2: All comments have been addressed

2. Does this manuscript meet PLOS Global Public Health’s [publication criteria](https://nam02.safelinks.protection.outlook.com/?url=https%3A%2F%2Ftrack.editorialmanager.com%2FCL0%2Fhttps%3A%252F%252Fjournals.plos.org%252Fglobalpublichealth%252Fs%252Fcriteria-for-publication%2F1%2F010f019c8e81d3a1-7317844b-b801-4d20-8b7e-55a84ad1a652-000000%2F_ULjYXKvSsqiv1wfJHTzFEjqezhuf0BcvlN9veAs-CY%3D251&data=05%7C02%7Cjsutto33%40jh.edu%7C300d10059b434aeb32ec08de7397e2cd%7C9fa4f438b1e6473b803f86f8aedf0dec%7C0%7C0%7C639075293338217403%7CUnknown%7CTWFpbGZsb3d8eyJFbXB0eU1hcGkiOnRydWUsIlYiOiIwLjAuMDAwMCIsIlAiOiJXaW4zMiIsIkFOIjoiTWFpbCIsIldUIjoyfQ%3D%3D%7C0%7C%7C%7C&sdata=FrzaJ5mKH1uZF%2F%2FLcAiS8hBD2oCIxo9tSDKu41gebyQ%3D&reserved=0)? Is the manuscript technically sound, and do the data support the conclusions? The manuscript must describe methodologically and ethically rigorous research with conclusions that are appropriately drawn based on the data presented.

Reviewer #1: Yes

Reviewer #2: Yes

3. Has the statistical analysis been performed appropriately and rigorously?

Reviewer #1: Yes

Reviewer #2: Yes

4. Have the authors made all data underlying the findings in their manuscript fully available (please refer to the Data Availability Statement at the start of the manuscript PDF file)?

The [PLOS Data policy](https://nam02.safelinks.protection.outlook.com/?url=https%3A%2F%2Ftrack.editorialmanager.com%2FCL0%2Fhttps%3A%252F%252Fjournals.plos.org%252Fglobalpublichealth%252Fs%252Fdata-availability%2F1%2F010f019c8e81d3a1-7317844b-b801-4d20-8b7e-55a84ad1a652-000000%2FKAn8ezuyw-HJEvLFUElOoRCF2787GdLqWN-oMAiRx0k%3D251&data=05%7C02%7Cjsutto33%40jh.edu%7C300d10059b434aeb32ec08de7397e2cd%7C9fa4f438b1e6473b803f86f8aedf0dec%7C0%7C0%7C639075293338228965%7CUnknown%7CTWFpbGZsb3d8eyJFbXB0eU1hcGkiOnRydWUsIlYiOiIwLjAuMDAwMCIsIlAiOiJXaW4zMiIsIkFOIjoiTWFpbCIsIldUIjoyfQ%3D%3D%7C0%7C%7C%7C&sdata=igziBcI5cxc96ZKDCxLzYOivM%2BgFaUtwmU9CkJX3%2Buo%3D&reserved=0) requires authors to make all data underlying the findings described in their manuscript fully available without restriction, with rare exception. The data should be provided as part of the manuscript or its supporting information, or deposited to a public repository. For example, in addition to summary statistics, the data points behind means, medians and variance measures should be available. If there are restrictions on publicly sharing data—e.g. participant privacy or use of data from a third party—those must be specified.

Reviewer #1: No

Reviewer #2: Yes

**RESPONSE: We previously uploaded the data to Data.CDC.Gov with the title “Benin_GANC_BehavioralData.” URL:** [**https://data.cdc.gov/Global-Health/Benin_GANC_BehavioralData/8592-cnvk/about_data.**](https://data.cdc.gov/Global-Health/Benin_GANC_BehavioralData/8592-cnvk/about_data.)

5. Is the manuscript presented in an intelligible fashion and written in standard English?

PLOS Global Public Health does not copyedit accepted manuscripts, so the language in submitted articles must be clear, correct, and unambiguous. Any typographical or grammatical errors should be corrected at revision, so please note any specific errors here.

Reviewer #1: Yes

Reviewer #2: Yes

6. Review Comments to the Author

Please use the space provided to explain your answers to the questions above. You may also include additional comments for the author, including concerns about dual publication, research ethics, or publication ethics. (Please upload your review as an attachment if it exceeds 20,000 characters)

Reviewer #1: Thank you for addressing the majority of reviewer comments and for responding to each reviewer's comments. There are a few topics that I still believe require some minor revisions:

-I see that you site the parent study once in the introduction, but I would cite it again in the methods to make it easier for readers to reference.
-I think your move to only have two aims does improve clarity and the aims are stated clearly in 89-94. However, there is still some confusion in how these aims are discussed throughout the paper. For example, I *think* the first few paragraphs of the results address your first aim. However, Table 2 is referenced and is located in this section and Table 2 seems to provide results related to Aim 2.
-The results for Aim 2 are very clear and comprehensive, but I am not sure what the qualitative results are for Aim 1. Explicit themes are not highlighted. I would include a subheading at the start of the results section to reference acceptability of group care, and then clearly present themes and any quantitative data you have to support acceptability. So inclusion and discussion of Table 3, which I think goes with your first aim, would be moved up. Then you can move to discussion of Aim 1 qual and quant results

**RESPONSE: Thank you for the suggestions.**

- **We have added more explicit references to the parent study under Results and expanded on the cRCT results.**
- **We have reorganized our Results section to present results pertaining to the first aim before moving on to those pertaining to the second aim.**
  - **We have added the sub-heading “Acceptability of Group Care.”**
  - **At the same time, many Results interrelate to each other and to both aims, so we also added another sub-heading “Survey Data on Acceptability, Self-Efficacy, and Comparison to Qualitative Findings.”**
  - **We realize that this does not completely separate the results by study aim, but this struck us as the best approach to make Results easier to follow.**
- **We have moved the Tables you mention to reinforce the newly reorganized text.**

Reviewer #2: Thank you for the opportunity to review this manuscript - it does add value add to the literature and is is very close to ready. I appreciate how responsive the authors were to the reviewers.

Minor suggestions:

1. The theory of change is discussed briefly in the introduction, but it really is a guiding principle according the 2020 article as well as the actual theory of change article. It is considered on the causal pathway. A touch more about how this is foundational to the present study and results is needed since, it seems, this the first to actually pull in self-efficacy. I appreciate how self-efficacy, through the TOC, is directly linked into table 2 and revisited in the discussion. The introduction could use a touch more positioning of the 2020 and TOC articles since these are foundational and at the core of this paper (Appendix 4 is great and really helpful for context. Suggestion: Number the appendices in order of presentation in the manuscript; i.e., Appendix 4 is the first one cited in the introduction. At the same time, Bandura is overexplained and could be tightened up to improve flow and overall reading.

**RESPONSE: In the Introduction, we have removed the excessive explanation of “self-efficacy” vs. “outcome efficacy” in the Bandura framework and gave more emphasis to how the Grenier et al theory of change places self-efficacy on a causal pathway to behavior change. Additionally, we have added a new citation to the Disussion section (Mehay et al 2024) for contrast, as it presents another conceptualization of self-efficacy as an outcome of G-ANC processes, rather than a mechanism of G-ANC.**

2. Typo/grammar: Line 102 left an incomplete phrase, "Every woman was" and the sentence on Line 104 - "interacted as a group" is odd phrasing give it is a participatory session.

**RESPONSE: We have corrected the incomplete phrase; thanks to your observation, we realized parts of lines 106-112 had been truncated or transposed and were able to adjust the text.**

3. Table 2 is nice, but since it seems it includes the same quotes that are in the text, so then it feels redundant. Given multiple speakers are presented in column 3, it is nearly unreadable. The data are not useful to readers in this format. Illustrative quote tables need to provide information that the text does not; so it seems it could be deleted since no new information is coming from it.

**RESPONSE: We have improved readability by adding space between the different quotes in Table 2. We feel the table adds new information because it defines the self-efficacy domains that emerged as themes when we were analyzing the focus group transcripts, and lines them up with sample quotes. Nowhere else in the manuscript have we explained the concept of each self-efficacy domain and it is important for the reader to have a clear delineation of each one.**

4. Domain 1: Emergency care-seeking: The first two illustrative quotes do not seem to fit with an emergency decision, but rather about timing and going to a health facility (domain #4). In part, the final analysis to group into domains feels incomplete or forced for the emergency domain (the third quote perfectly aligns). Are there more to the quotes to show its referring to emergencies arising because as written just seems to be about going into labor and not an emergency situation.

**RESPONSE: This point is well taken; in reviewing the transcripts and our definition of the Emergency Care-Seeking domain, we have realized that our working definition was much broader and the events being considered did not align with “emergency” as it is defined in the maternal health literature. We have therefore re-named this self-efficacy domain “Timely Care-Seeking” through the manuscript.**

4. Domain 2: The Pregnancy Management domain quotes are difficult for readers unfamiliar with cultural aspect of the leaf water, so it is unclear what the person is saying about the leaf water as self-care.

**RESPONSE: While we recognize that the phrasing “leaf water” is culturally specific, we believe that our existing use of brackets with interpreter notes makes it clear that this is a reference to “medicinal” plant water or traditional medicine taken to relieve pregnancy symptoms.**

5. Domain 4: Facility Delivery: These quotes seem like they are related to birth preparedness (see Ghana studies) and also advocacy aspects related to when to go to a facility. It seems that quotes can fall into multiple categories, but then it makes the domain names feel slightly off. Domains 1 and 4 seem to capture seeking care.

**RESPONSE: Thanks for this thoughtful observation. We agree that many quotes could fall into multiple categories. We see how both Domains 1 and 4 could be understood as relating to the first of the three delays contributing to maternal mortality (timely care-seeking). The difference between Domains 1 and 4 is that the first domain includes behaviors like initiating antenatal care early in one’s pregnancy and seeking care promptly in the presence of pregnancy danger signs, while the fourth domain is strictly related to the behavior of choosing a facility delivery and believing in one’s ability to assert that decision. This potential confusion is the reason why we have chosen to keep Table 2, as it includes a second column with definitions of each domain, “Definition (Confidence that one can…).”**

6. Lines 240-242, vicarious: the selected quote does not really show that there is learning from others experiences at work.

**RESPONSE: We selected this quote to show structural evidence that the group setting enables observational learning. However, your comment made us reflect on the strength of this example and we have replaced it with a more directly relevant quote: “Sometimes they put on theater [role play], and we watch it as an example played out between the midwife and the Yovonon [nickname for a health aide who takes care of the delivery].” We have retained the other quote under the Results sub-section “Acceptability of Group Care.”**

7. I assume formatting of tables and appendices will happen at the copy edit phase.

**RESPONSE: We understand this assumption to be correct.**

8. Discussion - linkages of domains and concepts to other published results like was done for IPTp is needed to connect to other studies in LMICs (e.g., reflecting knowledge change, empowerment, birth preparedness and complication readiness, preference for G-ANC, etc.) would strengthen the self-efficacy contribution. It helps with the logic argument because it would show that the innovative analysis and results from Benin, resonate with other studies that had similar outcomes but did not frame this as self-efficacy. In other words, there is strong argument that this first article to approach it in this way, contribute general knowledge by labeling it as such.

**RESPONSE:**

**We added a new paragraph reflecting on the conceptual difference between the way in which Mehay et al, 2024 placed self-efficacy as an outcome of G-ANC in the logic model resulting from their systematic reviews, rather than as a mechanism on the causal pathway as Grenier et al does.**

**“A realist evaluation on the mechanisms of effect for group models of antenatal care produced a Pregnancy Circles Logic Model with increased self-efficacy occupying a prominent place in the model, and identified as one of six intermediate outcomes in the model (21). The intermediate outcomes result in turn from six mechanisms of change including group cohesiveness, health literacy, and psychological empowerment (21). A scoping review on group antenatal care for adolescents identified empowerment as a key theme, and self-efficacy as one contributor to empowerment (22). This scoping review in turn identified two studies that highlighted self-efficacy as a mechanism for the effects of G-ANC in adolescents (23, 24). In many other studies including in Nigeria (25) and Ghana (26), self-efficacy is not reported in the factors affecting effectiveness (26), or is not present in the theory of change as either a mechanism or an outcome (25).”**

**21. Mehay A, Motta GD, Hunter L, Rayment J, Wiggins M, Haora P, et al. What are the mechanisms of effect of group antenatal care? A systematic realist review and synthesis of the literature. BMC Pregnancy Childbirth. 2024;24(1):625.**

**22. Lanyo TN, Lori JR. Identifying health outcomes and future research directions of group antenatal care among adolescents in low and middle-income countries: a scoping review. BMJ Glob Health. 2025;10(3).**

**23. Malchi F, Abedi P, Iravani M, Mohammadi E, Saadati N, Maraghi E. Perception of Group Prenatal Care among Pregnant Adolescents: A Qualitative Study in Iran. J Pediatr Adolesc Gynecol. 2023;36(4):413-9.**

**24. Vandermorris A, McKinnon B, Sall M, Witol A, Traore M, Lamesse-Diedhiou F, et al. Adolescents' experiences with group antenatal care: Insights from a mixed-methods study in Senegal. Trop Med Int Health. 2021;26(12):1700-8.**

**25. Evans WD, Ochu CL, Bingenheimer JB, Adebayo SB, David FA, Gar SA, et al. Implementation and Baseline Evaluation of an Evidence-Based Group Antenatal Care Program in Two Nigerian States. Int J Environ Res Public Health. 2024;21(11).**

**26. Kukula VA, Awini E, Ghosh B, Apetorgbor V, Zielinski R, Amankwah G, et al. Effect of group antenatal care versus individualized antenatal care on birth preparedness and complication readiness: a cluster randomized controlled study among pregnant women in Eastern Region of Ghana. BMC Pregnancy Childbirth. 2024;24(1):546.**

Overall, this is an improved version and nearly ready for publication. The authors were attentive to reviewers’ concerns.

7. PLOS authors have the option to publish the peer review history of their article ([what does this mean?](https://nam02.safelinks.protection.outlook.com/?url=https%3A%2F%2Ftrack.editorialmanager.com%2FCL0%2Fhttps%3A%252F%252Fjournals.plos.org%252Fglobalpublichealth%252Fs%252Feditorial-and-peer-review-process%2523loc-peer-review-history%2F1%2F010f019c8e81d3a1-7317844b-b801-4d20-8b7e-55a84ad1a652-000000%2FNFjayrZfvS_CwJMacefHUfuOjxLI_okkxIDvkORnuxA%3D251&data=05%7C02%7Cjsutto33%40jh.edu%7C300d10059b434aeb32ec08de7397e2cd%7C9fa4f438b1e6473b803f86f8aedf0dec%7C0%7C0%7C639075293338237937%7CUnknown%7CTWFpbGZsb3d8eyJFbXB0eU1hcGkiOnRydWUsIlYiOiIwLjAuMDAwMCIsIlAiOiJXaW4zMiIsIkFOIjoiTWFpbCIsIldUIjoyfQ%3D%3D%7C0%7C%7C%7C&sdata=gUhMc6VGB1gHMHcGfZ2OQkTLTKJOn6LU7JBELBpL1fw%3D&reserved=0)). If published, this will include your full peer review and any attached files.

**Do you want your identity to be public for this peer review?** If you choose “no”, your identity will remain anonymous but your review may still be made public.

For information about this choice, including consent withdrawal, please see our [Privacy Policy](https://nam02.safelinks.protection.outlook.com/?url=https%3A%2F%2Ftrack.editorialmanager.com%2FCL0%2Fhttps%3A%252F%252Fwww.plos.org%252Fprivacy-policy%2F1%2F010f019c8e81d3a1-7317844b-b801-4d20-8b7e-55a84ad1a652-000000%2Fui6EVy6xN2wGVJPa40RyPxE5kf5WYdWgYZ-0nUw3tbA%3D251&data=05%7C02%7Cjsutto33%40jh.edu%7C300d10059b434aeb32ec08de7397e2cd%7C9fa4f438b1e6473b803f86f8aedf0dec%7C0%7C0%7C639075293338246304%7CUnknown%7CTWFpbGZsb3d8eyJFbXB0eU1hcGkiOnRydWUsIlYiOiIwLjAuMDAwMCIsIlAiOiJXaW4zMiIsIkFOIjoiTWFpbCIsIldUIjoyfQ%3D%3D%7C0%7C%7C%7C&sdata=lozcyystDJfMwqCXARg967diL2bE6oF5AaUz2B3rA7k%3D&reserved=0).

Reviewer #1: No

Reviewer #2: No
